# Supplementary material for: Predicting visual function by interpreting a neuronal wiring diagram
Source: Nature. 2024 Oct 2;634(8032):113–23. doi: 10.1038/s41586-024-07953-5 (PMC11446822; doi:10.1038/s41586-024-07953-5)
Supplement: Supplementary file 6 — CRF and ERF predictions for individual TmY4 and TmY9 cells. Analogous to Supplementary Data 3, but for TmY target types. Shown are the top four monosynaptic pathways, the strongest pathway passing through each of the top ten intermediary types (ranking from Extended Data Fig. 7), and the trisynaptic pathway Tm1–TmY–Dm3–TmY (see the section entitled Prediction of spatial normalization). [file 41586_2024_7953_MOESM6_ESM.zip › DataS4/TmY9qΓèÑ/720575940627511695.pdf]

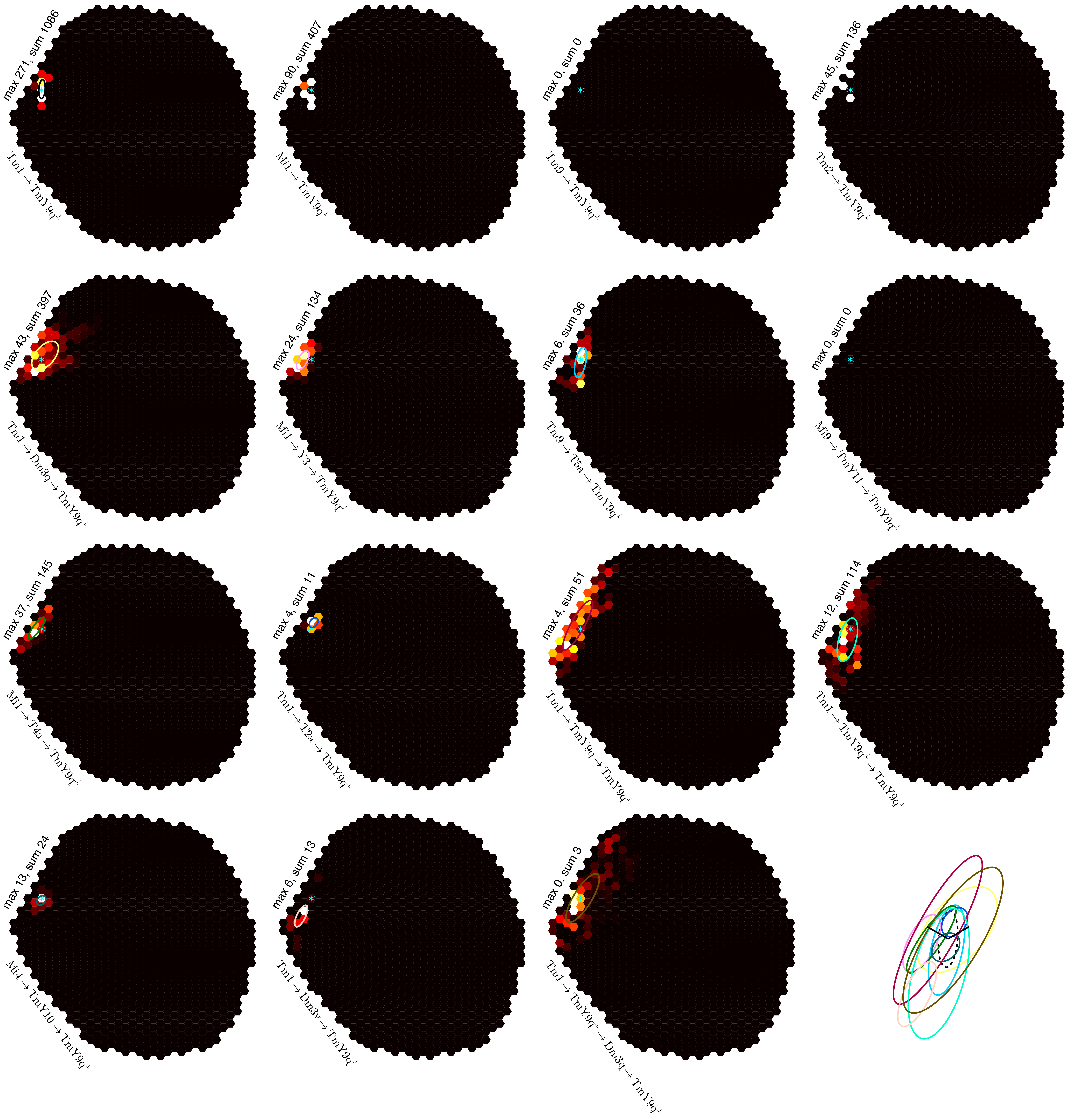
max 271, sum 1086
Tm1 → TmY9q⊥
max 90, sum 407
Mi1 → TmY9q⊥
max 0, sum 0
Tm9 → TmY9q⊥
max 45, sum 136
Tm2 → TmY9q⊥
max 43, sum 397
Tm1 → Dm3q → TmY9q⊥
max 24, sum 134
Mi1 → Y3 → TmY9q⊥
max 6, sum 36
Tm9 → T5a → TmY9q⊥
max 0, sum 0
Mi9 → TmY11 → TmY9q⊥
max 37, sum 145
Mi1 → T4a → TmY9q⊥
max 4, sum 11
Tm1 → T2a → TmY9q⊥
max 4, sum 51
Tm1 → TmY9q → TmY9q⊥
max 12, sum 114
Tm1 → TmY9q⊥ → TmY9q⊥
max 13, sum 24
Mi4 → TmY10 → TmY9q⊥
max 6, sum 13
Tm1 → Dm3v → TmY9q⊥
max 0, sum 3
Tm1 → TmY9q⊥ → Dm3q → TmY9q⊥
